# Alpha-Asaronol Alleviates Dysmyelination by Enhancing Glutamate Transport Through the Activation of PPARγ-GLT-1 Signaling in Hypoxia-Ischemia Neonatal Rats

**DOI:** 10.3389/fphar.2022.766744

**Published:** 2022-03-23

**Authors:** Yuhang Ge, Fei Zhen, Ziqi Liu, Zhaowei Feng, Gui Wang, Chu Zhang, Xingqi Wang, Ying Sun, Xiaohui Zheng, Yajun Bai, Ruiqin Yao

**Affiliations:** ^1^ Department of Cell Biology and Neurobiology, Xuzhou Key Laboratory of Neurobiology, Xuzhou Medical University, Xuzhou, China; ^2^ Department of Human Anatomy, Xuzhou Medical University, Xuzhou, China; ^3^ Hongze Huaian District People’s Hospital, Hongze, China; ^4^ Key Laboratory for Biotechnology on Medicinal Plants of Jiangsu Province, School of Life Science, Jiangsu Normal University, Xuzhou, China; ^5^ Key Laboratory of Synthetic and Natural Functional Molecule Chemistry of the Ministry of Education, College of Chemistry and Materials Science, Northwest University, Xi’an, China

**Keywords:** *α*-asaronol, glutamic acid, PPARγ, white matter injury, oligodendrocyte precursor cells

## Abstract

Preterm white matter injury (PWMI) is the most common form of brain damage in premature infants caused by hypoxia-ischemia (HI), inflammation, or excitotoxicity. It is characterized by oligodendrocyte precursor cell (OPC) differentiation disorder and dysmyelination. Our previous study confirmed that alpha-asarone (α-asaronol), a major compound isolated from the Chinese medicinal herb *Acorus gramineus* by our lab, could alleviate neuronal overexcitation and improve the cognitive function of aged rats. In the present study, we investigated the effect and mechanism of α-asaronol on myelination in a rat model of PWMI induced by HI. Notably, α-asaronol promoted OPC differentiation and myelination in the corpus callosum of PWMI rats. Meanwhile, the concentration of glutamate was significantly decreased, and the levels of PPARγ and glutamate transporter 1 (GLT-1) were increased by α-asaronol treatment. *In vitro,* it was also confirmed that α-asaronol increased GLT-1 expression and recruitment of the PPARγ coactivator PCG-1a in astrocytes under oxygen and glucose deprivation (OGD) conditions. The PPARγ inhibitor GW9662 significantly reversed the effect of α-asaronol on GLT-1 expression and PCG-1a recruitment. Interestingly, the conditioned medium from α-asaronol-treated astrocytes decreased the number of OPCs and increased the number of mature oligodendrocytes. These results suggest that α-asaronol can promote OPC differentiation and relieve dysmyelination by regulating glutamate levels *via* astrocyte PPARγ-GLT-1 signaling. Although whether *α*-asaronol binds to PPARγ directly or indirectly is not investigated here, this study still indicates that *α*-asaronol may be a promising small molecular drug for the treatment of myelin-related diseases.

## Highlights

α-Asaronol promotes OPC differentiation and alleviates dysmyelination.
α-Asaronol decreases the concentration of glutamate in the brains of PWMI model rats.
α-Asaronol increases the expression of GLT-1 and PPARγ in astrocytes.
The promyelination effect of α-asaronol is due to glutamate uptake mediated by PPARγ-GLT-1 signaling.

## Introduction

Premature white matter injury (PWMI) is the leading cause of child death worldwide and has become a global health priority. Although the survival rate of premature infants has improved due to the increasing importance of developing a neonatal intensive care unit, survivors will have a high risk of life-long disabilities, including cerebral palsy, epilepsy, and impairment of cognition and behavior (Younge et al., 2017). During the development of the oligodendroglial lineage, oligodendrocyte precursor cells (OPCs) are predominant at the early stage with capabilities of migration, differentiation, and remyelination in the central nervous system (CNS) (Back et al., 2001), and mature oligodendrocytes are responsible for forming myelin membranes around axons, enabling fast saltatory nerve conduction and axon integrity protection. Due to immaturity of the periventricular vasculature, OPCs are intrinsically vulnerable to adverse milieu such as infection, inflammation, and hypoxia-ischemia (HI), resulting in proliferation and differentiation disorders and eventually causing white matter injuries (Back, 2017).

Glutamate-mediated excitotoxicity has been shown to cause abnormal white matter development or WMI in neonatal rodents. Neonatal mouse hyperoxia disrupts glutamate homeostasis, and high extracellular glutamate levels lead to immature oligodendroglia apoptosis and white matter maturation delay and eventually reduce white matter diffusivity in adults (Schmitz et al., 2011). Enhancement of extracellular glutamate uptake by upregulating glutamate transporters significantly reduced HI-induced PWMI (Yeh et al., 2017). Maintenance of extracellular glutamate homeostasis is based on functional sodium-dependent glutamate transporters. Five subtypes of excitatory amino acid transporters (EAATs) are designated EAAT1-EAAT5, which belong to the solute carrier 1 (SLC1) family of transmembrane amino acid transporters (Rose et al., 2018; Magi et al., 2019). Astrocytic excitatory amino acid transporter 2 (EAAT2), also known as glutamate transporter 1 (GLT-1), plays an important role in cleaning up to 95% of glutamate (Haugeto et al., 1996). Perinatal infection/inflammation and HI contribute to the depletion of adenosine triphosphate (ATP) in the preterm brain, leading to disruption of GLT-1 in both expression and reversal function, while glutamate uptake is an energy-dependent process (Murugan et al., 2013).

Recently, we isolated α-asaronol from the rhizome of *Acorus calamus* var. *angustatus* Besser (Bai et al., 2020). It is a metabolite of α-asarone, which has long been a traditional medicine for the treatment of cardio-cerebrovascular disease (Limón et al., 2009; Lam et al., 2017). Although studies have revealed that α-asarone exhibits neuroprotective effects on epilepsy (Pages et al., 2010; Huang et al., 2013), Alzheimer’s disease (Chen et al., 2020), and stroke (Lee et al., 2018), the nonnegligible side effects of its carcinogenic and genotoxic potential limit its application (Chamorro et al., 1993; Marczewska et al., 2013). With the synthesis of α-asaronol in our lab (Bai et al., 2019), its pharmacokinetics (Sun et al., 2019) and biological activities were gradually evaluated, such as antioxidative stress (He et al., 2018), alleviating neurotoxicity, and anticonvulsant (Jin et al., 2020) and anti-epilepsy by balancing neurotransmitters (data unpublished). However, the effect of α-asaronol on myelination in PWMI neonatal rats and the underlying mechanisms remain elusive.

In this study, we found that α-asaronol could effectively promote myelination and decrease the high level of glutamate triggered by HI in encephalic regions of postnatal pups. Meanwhile, GLT-1 expression was also increased in the model rats treated with α-asaronol. We predicted the potential target of α-asaronol using molecular docking analyses and further confirmed that α-asaronol regulates GLT-1 by activating peroxisome proliferator-activated receptor γ (PPARγ) by recruiting peroxisome proliferator-activated receptor-γ coactivator-1 alpha (PGC-1α) (Jin et al., 2020). Our study suggests that α-asaronol can relieve glutamate excitotoxicity and promote OPC differentiation and myelination by activating PPARγ-GLT-1 signaling in a rat model of PWMI.

## Materials and Methods

### Animals and Drug Treatment

Adult male and female Sprague–Dawley (SD) rats were obtained from the Center of Experimental Animals of Xuzhou Medical University and bred in a sterile animal barrier system. The rats were maintained with a normal diet under a 12:12 h light:dark cycle. Postnatal 3-day rats (n ≈ 210) were randomly divided into the sham, hypoxia-ischemia (HI + normal saline group (HI + NS), and HI + α-asaronol groups (HI + ASA). The PWMI neonatal rat model was established as described previously (Vannucci and Vannucci, 1997). Briefly, pups were anesthetized with 2.5% isoflurane (Shandong Keyuan Pharmaceutical Co., Jinan, Shandong Province, China). The right common carotid artery was isolated and ligated from distal and proximal ends. After the skin was sutured and disinfected, the pups were returned to their mother’s cage, allowed to recover for 2 h, and then exposed to 8% O_2_ (92% N_2_ saturation) at 37°C for 2 h. The sham group underwent the same procedures without ligation of the common carotid artery. The rats were exposed to room air after hypoxia. For α-asaronol treatment, pups were treated with different doses of α-asaronol (25 mg/kg, 50 mg/kg, 100 mg/kg) at 1 h after hypoxia, every 8 h, twice a day.

### Tissue Preparation

At P3 + 12 h, P3 + 4 d, and P3 + 7 d (time after surgery), pups from each group were anesthetized with 2.5% isoflurane and sacrificed. For high-performance liquid chromatography (HPLC), western blot, and real-time quantitative PCR (RT–qPCR) tests, all the tissue samples were picked up on an ice pack and stored at −70°C. For immunofluorescence, pups were intracardially perfused with saline followed by fixation with 4% cold paraformaldehyde and then postfixed in 4% paraformaldehyde for 6 h at 4°C followed by 15% and 30% sucrose until the water was replaced completely. All procedures in the experiment were consistent with Chinese legislation on the use and care of laboratory animals and were approved by the Xuzhou Medical University committee for animal experiments.

### Primary Cell Culture and Treatment

Primary mixed cells were isolated and cultured from the brains of P0∼P2 SD rats. Briefly, the cerebral cortex was separated, cut into 1 mm^3^ pieces, and centrifuged at 1,000 rpm for 2 min to remove the supernatant. Digestion of sediment was performed according to the instructions of the digestion kit, and then the digestion was terminated with DMEM/F-12 (1:1) medium (HyClone, United States) containing 10% fetal bovine serum (FBS, Gibco). The samples were centrifuged at 1,200 rpm for 5 min, and the supernatant was discarded. The cells were resuspended in DMEM/F-12 (1:1) medium and placed into a T25 culture flask at 1 × 10^6^ cells/cm^2^. The medium was changed every 2–3 days. Approximately 8–9 days after plating, the flasks were shaken at 200 rpm for 1 h on a cell shaker to remove microglial cells. After reincubation for 2–4 h at 37°C, the flasks were shaken at 200 rpm for 18 h, and then the supernatant was collected and centrifuged. Finally, OPCs were resuspended and plated onto coverslips in 24-well plates coated with poly-D-lysine (0.1 mg/ml, Sigma).

The astrocytes on the bottom of the flasks were digested with 0.25% trypsin and plated onto 100 mm Petri dishes. In order to observe the effect and mechanism of α-asaronol on GLT-1 expression, astrocytes (passages 3 to 7) were divided into normal culture group (control), glucose deprivation/reperfusion group (OGD/R) [astrocytes were washed with sterile phosphate-buffered saline (PBS), and incubated in glucose-free DMEM in an anaerobic incubator containing 95% N_2_ and 5% CO_2_ for 6 h at 37°C and then reoxygen for 12 h], OGD/R + α-asaronol group (astrocytes were pretreated with 1, 10, or 100 µM α-asaronol for 24 h before OGD), and OGD/R + α-asaronol + GW9662 group [astrocytes were pretreated with α-asaronol and 4 µM GW9662 (Meryer Chemical Technology Co., Ltd., Shanghai, China) for 24 h before OGD]. To clarify whether α-asaronol influences OPC differentiation by regulating GLT-1 expression and glutamate levels, a conditioned medium from the above four groups was used to treat OPCs for 3–5 days.

In this study, a conditioned medium from primary neurons was used to promote GLT-1 maturation of astrocytes *in vitro*. Briefly, the brains of P0∼P1-day-old SD rats were collected, and the hippocampi were isolated. After the hippocampus was cut into small pieces, 0.25% trypsin with 0.125% ethylene diamine tetraacetonitrile was added to digest the small pieces at 37°C for 15 min. Digestion was terminated with DMEM/F-12 (1:1) medium containing 10% FBS. After the cells were gently blown into single cells using a pipette, the suspension was filtered with a 200-mesh sieve and centrifuged at 1,200 rpm for 5 min. The precipitate was resuspended in PBS and centrifuged again. The supernatant was discarded, and the cells were resuspended in neurobasal-A medium (Gibco, 10888022) supplemented with cytarabine (Aladdin, 147-94-4, 5 μmol/L) and seeded into Petri dishes for 24 h. After washing with PBS, the cytarabine-free neurobasal-A medium was changed. The conditioned medium from neurons was collected after 7 days.

### Immunofluorescence Staining

For immunohistofluorescence, 20 μm serial cryosections were made and collected on polylysine-coated slides. For immunocytofluorescence, cells grown on coverslips were washed with 0.01 mol/L PBS and then fixed with 4% paraformaldehyde for 15 min at room temperature. After washing with PBS, brain sections or cells were blocked in PBS with 0.3% Triton X-100 and 10% goat serum for 1 h or 20 min at room temperature and then incubated with primary antibody at 4°C overnight and secondary antibody for 2 h at room temperature. Both antibody incubations were followed by washing with PBS. Finally, 2-(4-amidinophenyl)-6-indolecarbamidine (DAPI, Beyotime, C1005) was used to label nuclei. Staining specificity was assessed by omitting the primary antibody. Quantitative analysis of immunofluorescence staining was performed using ImageJ software (NIH, Bethesda, MD, United States). Primary and secondary antibodies were as follows: rabbit oligodendrocyte lineage transcription factor 2 (Olig2, Proteintech, 13999-1-AP, 1:200), rabbit glial fibrillary acidic protein (GFAP, Proteintech, 16825-1-AP, 1:400), mouse GLT-1 (Santa Cruz, sc-365634, 1:200), rabbit GLT-1 (Abcam, ab41621, 1:400), mouse GLT-1 (Santa Cruz, sc-365634, 1:100), mouse myelin basic protein (MBP, Santa Cruz, sc-271524, 1:500), mouse Protein APC (CC1, Invitrogen, MA1-25884, 1:50), rabbit platelet-derived growth factor receptor-alpha (PDGFRα, Abcam, ab203491, 1:400), rabbit ATPase, Na^+^/K^+^ transporting, alpha 1 polypeptide (ATP1α1, Proteintech, 14418-1-AP, 1:500), goat anti-mouse IgG (H + L) Alexa Fluor ®555-conjugated, or goat anti-rabbit IgG (H + L) Alexa Fluor ®555 (1:500, Cell signaling technology).

### Western Blot

The ipsilateral hemispheres of the rats were dissected and homogenized at P3 + 12 h, P3 + 4 d, and P3 + 7 d after surgery. The homogenates were centrifuged at 12,000 rpm for 20 min at 4°C. The concentration of protein in the supernatant was determined using bicinchoninic acid (Beyotime, China). The protein samples (20 µL) were separated by sodium dodecyl sulfate–polyacrylamide gel electrophoresis (SDS–PAGE) and then transferred to nitrocellulose membranes (Millipore Corporation, MA, United States). The membranes were blocked with 5% nonfat milk at room temperature for 2 h, followed by overnight incubation at 4°C with anti-PDGFRα antibody (Abcam, ab203491, 1:500), anti-MBP antibody (Santa Cruz, sc-271524, 1:500), anti-GLT-1 antibody (Santa Cruz, sc-365634, 1:1,000), anti-ATP1α1 antibody (Proteintech, 14418-1-AP, 1:5,000), anti-PPARγ antibody (Proteintech, 22061-1-AP, 1:500), anti phospho-AMPA receptor 2 (GluA2) (Tyr876) antibody (p-GluR2, CST 4027s, 1:1,000), anti-GluR2 antibody (CST 13607s, 1:1,000), and anti-β-actin antibody (Proteintech, 20536-1-AP, 1:2000). After washing, the membranes were incubated with goat anti-mouse or goat anti-rabbit secondary antibodies (Abcam, ab216776 and ab216773, 1:5,000) for 2 h at room temperature. The bands on the membrane were scanned with an Odyssey infrared scanner (LI-COR Biosciences, Lincoln, NE, United States), and the density of the bands was analyzed with ImageJ software.

### Immunoprecipitation (Co-IP)

For PPAR-γ activation analysis, the astrocytes were collected and homogenized in 1/3 (w/v) ice-cold lysis buffer. Equal amounts of protein (500 μg) were precleared using protein A-Sepharose beads (40 μL) for 1 h at 4°C and then incubated with 5 μL of rabbit polyclonal anti-PPARγ antibody (Proteintech, 16643-1-AP) or mouse monoclonal anti-IgG (Cell Signaling Technology, Danvers, MA). The immune complex was affinity-precipitated with protein A-Sepharose beads and washed six times with 25 mM buffer (pH 7.4) containing 10 mM MgCl_2_, 1 mM NaF, 1% NP-40, and 1 mM Na_3_VO_4_. The immunoprecipitates were separated by SDS–PAGE. The levels of PGC-1α and histone deacetylase 3 (HDAC3) were analyzed by western blot using rabbit polyclonal anti-PGC-1α antibody (Novus, NBP1-04676, 1:1,000) and mouse anti-HDAC3 antibody (Santa Cruz, sc-376957, 1:1,000).

### High-Performance Liquid Chromatography

The ipsilateral hemispheres were isolated from the rats after HI for different times (P3 + 12, P3 + 4 d, P3 + 7 d). Tissue samples were treated with a mixture of methanol and distilled water (1:1) at a ratio of 100 mg/ml. The homogenate was centrifuged at 12,000 rpm for 20 min at 4°C, and the supernatant was collected. The sediment was added to ice-cold acetonitrile and homogenized and centrifuged. The supernatant was collected, and the pellet was resuspended, homogenized, and centrifuged again. The supernatant from the first and second centrifugation was mixed and dried with a rotary evaporator, and the content was dissolved in 80 µL distilled water for derivation. Standards and samples were mixed with 80 µL of 0.1 mM borate saline buffer (pH = 8.6), 160 µL distilled water, and 80 µL of 4 mM 9-fluorenylmethyl chloroformate (Fmoc-Cl) in a 40°C bath for 5 min for derivation. The derived product was filtered with a 0.22 µm Millipore filter membrane for chromatographic detection. The chromatographic analysis was performed using a Waters e2695 (Waters, United States) and UV detector at a wavelength of 265 nm. The analytical column (Hypersil ODS2, 260 × 4.0 mm, 5 µm particle size, Elite, China) was maintained at 30°C. The flow rate was set at 1 ml/min, and elution was performed using a linear gradient of mobile phases A (50 mM sodium acetate containing 1% tetrahydrofuran, pH = 4.8) and B (acetonitrile). HPLC was also used to detect the concentration of glutamate in the medium from the cultured astrocytes.

### Cell Viability Analysis

The CCK-8 (Beyotime Biotechnology Inc., Shanghai, China) assay was carried out to estimate cell viability. Primary astrocytes were seeded into 96-well plates at a density of 4 × 10^3^ cells per well and cultured for 2 days. After incubation with a fresh medium containing 1, 10, or 100 µM α-asaronol for 24 h, the cells were subjected to OGD for 6 h and reoxygenation for 12 h. Astrocytes were incubated with CCK-8 reagent for 1 h in the dark (5% CO_2_ at 37°C). The optical density (OD) value was measured at a wavelength of 450 nm on a microplate reader (BioTek, United States).

### Real-Time Quantitative PCR

RT-qPCR was used to investigate the transcript levels of GLT-1 and PPARγ. Total RNA from the pups (P3 + 12 h, P3 + 4 d, P3 + 7 d) from each group was extracted using TRIzol reagent (Invitrogen, United States). Extracted RNA was reverse-transcribed into cDNA using HiScript® II Q RT Super Mix for qPCR (Vazyme, China). RT-qPCR was performed using SYBR Green qPCR Master Mix (MCE, United States) and Roche Light Cycler 480 II (Roche, Switzerland). β-Actin was used as an internal standard. The relative gene expression was calculated according to the 2^−∆∆Ct^ method. The primers were as follows: GLT-1 (forward, GAA​GAC​ATC​CCG​TTC​ACA​AGA; reverse, TGA​TGC​TTT​ATC​CCC​ACA​GAC) and PPARγ (forward, GTT​CAA​GGA​CGG​GAT​GAA​TGT​CTT​A; reverse, CAT​CAG​CTT​GGC​CTG​CTC​AC).

### Molecular Docking Simulation

Molecular modeling was performed using Sybyl-x 2.0 software from Tripos Inc. (St. Louis, MO). The PPARγ structure used for docking was obtained from the Protein Data Bank (PDB, http://www.rcsb.org; PDB ID, 5Y2T). The cocrystalized ligand and water molecules were removed from the structure, while H atoms were added and side chains were fixed during protein preparation. The 3D structure of α-asaronol was constructed and energy-minimized using the MM2 molecular mechanics method with Chem3D Pro 14.0 (Cambridge Soft Co., United States). Before docking, PPARγ and α-asaronol were energetically minimized by the CHARMm force field. After preparation, the Surflex-Dock (SFXC) docking mode was performed using the default settings. Surflex-Dock was employed for the molecular docking study. Surflex-Dock scores (total scores) represent binding affinities.

### Statistical Analysis

All statistical analyses were performed with GraphPad Prism 7.0 (GraphPad Software; CA, United States), and the data were analyzed by an investigator blinded to the procedure using one-way analysis of variation (ANOVA) followed by either the Newman–Keuls or Tukey’s honestly significant difference post hoc test for comparisons among multiple groups. The data were expressed as the mean ± standard error of the mean (SEM). *p* < 0.05 was considered as significant.

## Results

### α-Asaronol Improves Oligodendrocyte Precursor Cell Differentiation and Relieves Dysmyelination in Preterm White Matter Injury Rats

**FIGURE 1:**
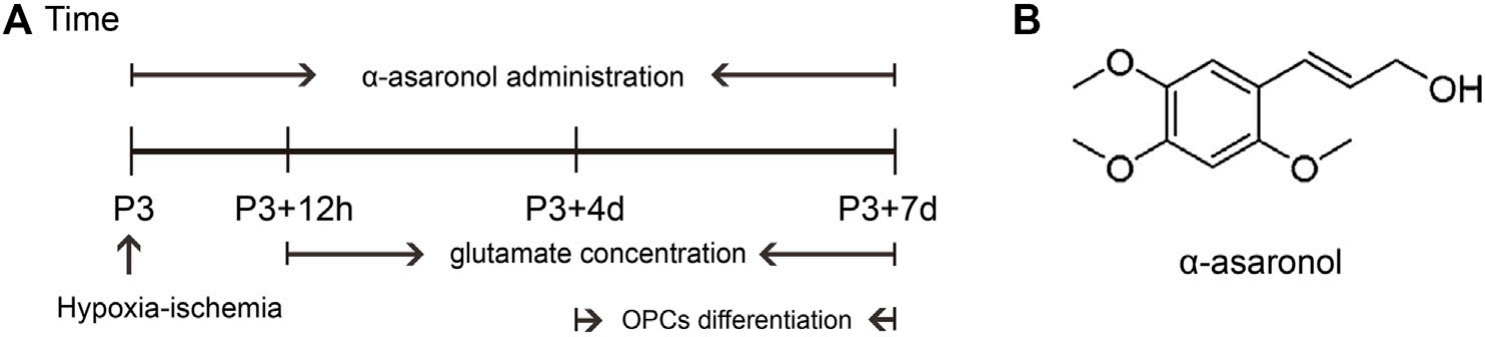
Schematic diagram of the experimental design *in vivo*. **(A)** Schedule of animal experiments. **(B)** Molecular structure formula of α-asaronol.

**FIGURE 2:**
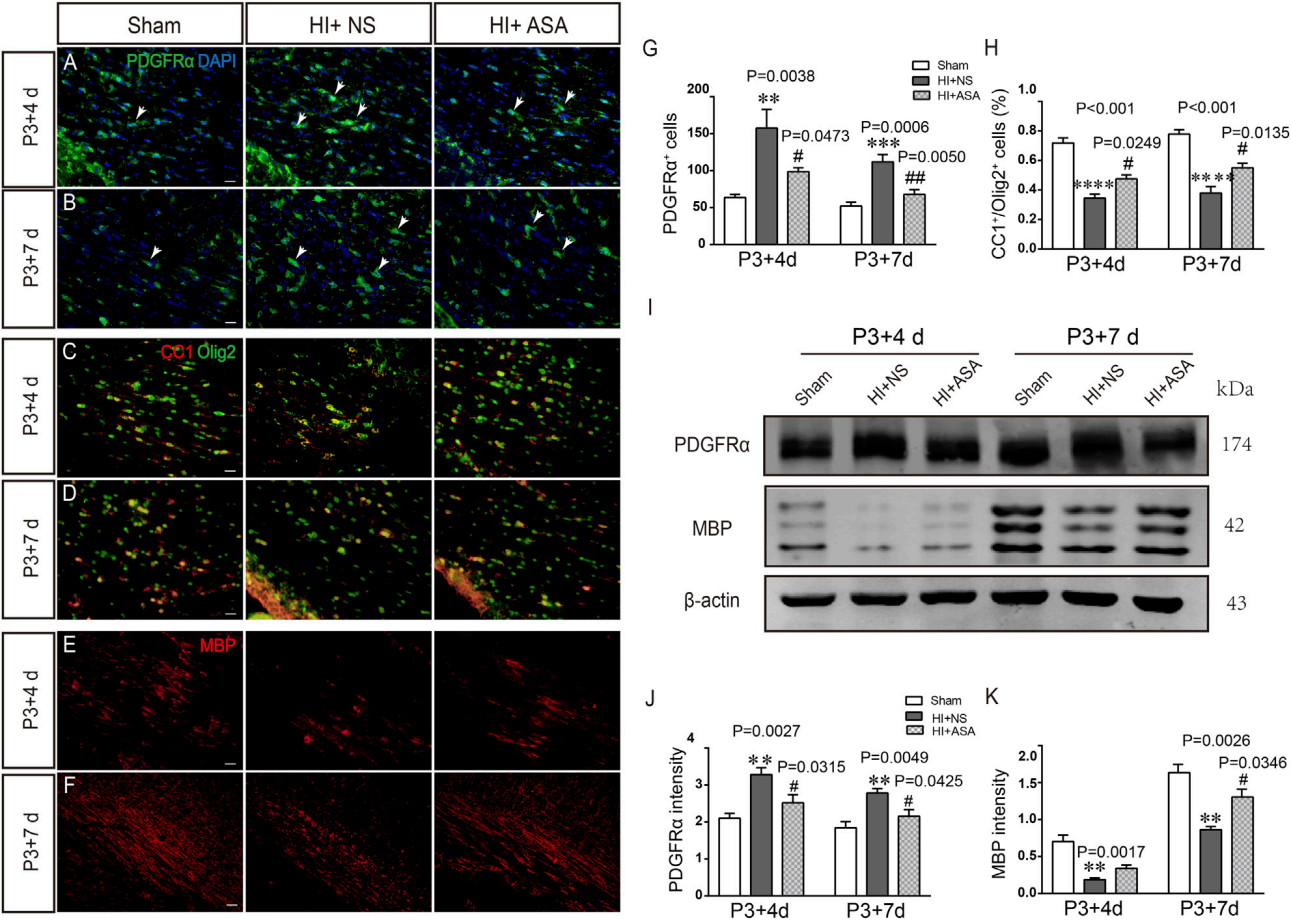
*α*-Asaronol improves OPC differentiation and relieves dysmyelination in PWMI models. Representative immunofluorescence images for PDGFRα-labeled OPCs. **(A,B)** CC1-labeled mature oligodendrocyte and Olig2-labeled oligodendrocyte lineage **(C,D)** and MBP-labeled myelin sheath **(E,F)** in the corpus callosum at P3 + 4 d and P3 + 7 d after HI. Scale bar, 20 µm. **(G,H)** Quantification of the number of PDGFRα^+^ cells and the percentage of CC1+/Olig2+ cells. **(I,K)** Western blotting was performed to analyze PDGFRα and MBP protein levels. All data are expressed as the mean ± SEM. **p < 0.01, ***p < 0.001, ****p < 0.001 vs. sham group. ^*#*^ *p <* 0.05, ^*##*^ *p* < 0.01 vs. HI + NS group. *N* = 4 for each group.

Overactivation of glutamate receptors causes calcium overload and is a major factor in OPC differentiation inhibition and cell death. To observe the effect of α-asaronol on OPC differentiation and myelination in a PWMI rat model, we designed an *in vivo* experiment. A schematic diagram of the experiment and the molecular structure formula of α-asaronol are shown in Figure 1. Immunofluorescence staining and quantitative analysis revealed that the number of PDGFRα^+^ OPCs in the corpus callosum in the NS group was much higher than that in the sham group, while α-asaronol significantly decreased the PDGFRα^+^ cell number at P3 + 4 d and P3 + 7 d (Figures 2A,B,G). In contrast, MBP-labeled myelin density and the ratio of CC1-labeled mature oligodendrocytes to Olig2-labeled OPCs/oligodendrocytes in the NS group were significantly lower than those in the sham group, but α-asaronol treatment obviously enhanced the ratio of CC1^+^/Olig2^+^ cells (Figures 2C,D,H) and MBP^+^ myelin density (Figures 2E,F). Protein analysis also showed that α-asaronol decreased PDGFR*α* levels and enhanced MBP levels (Figures 2I–K).

### α-Asaronol Decreases Glutamate Levels in the Brains of Preterm White Matter Injury Rats

**FIGURE 3:**
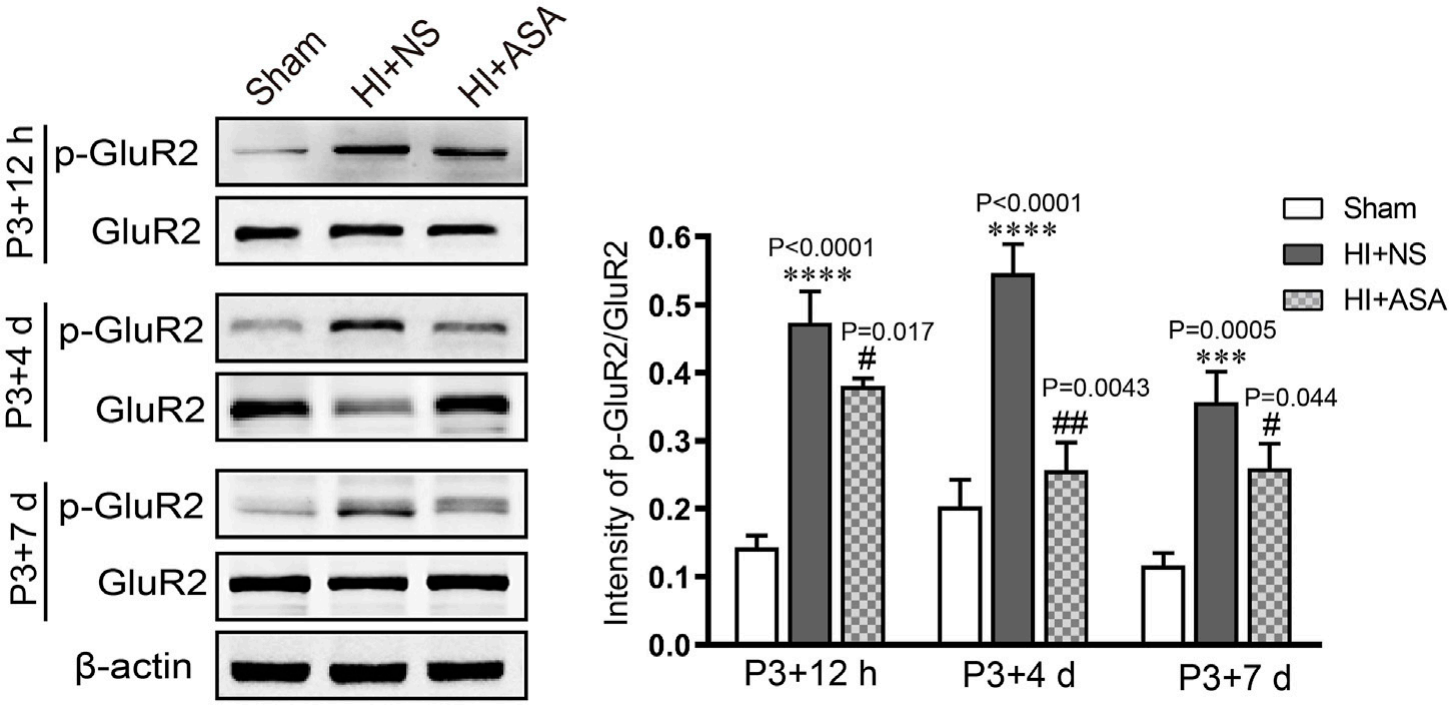
*α*-Asaronol inhibits HI-induced activation of the ionotropic glutamate receptor AMPAR. The protein level of p-GluR2 (subunit 2 of AMPAR) was detected by western blot at P3 + 12 h, P3 + 4 d, and P3 + 7 d after HI. HI induced an obvious increase in p-GluR2 (Tyr876), and α-asaronol (50 mg/kg) treatment decreased the level of p-GluR2. All data are expressed as the mean ± SEM. ***p < 0.001, ****p < 0.0001 vs. sham. ^*#*^ *p* < 0.05, ^*##*^ *p* < 0.01 vs. HI + NS. *N* = 4 for each group.

In order to investigate the effect of α-asaronol on glutamate levels, the samples were collected from the acute to chronic phase after HI, and the concentration of glutamate was detected by HPLC. The standard curve of glutamate was established. The linear range was from 1 μg/ml to 200 μg/ml, and the value of the correlation coefficient (r) was 0.9996. The glutamate concentration was substantially increased in the NS group at P3 + 12 h (0,341 ± 0.029 μg/mg), P3 + 4 d (0.402 ± 0.076 μg/mg), and P3 + 7 d (0.364 ± 0.007 μg/mg) compared with the sham group (0.198 ± 0.019 μg/mg for P3 + 12 h, 0.176 ± 0.014 μg/mg for P3 + 4 d; 0.174 ± 0.019 μg/mg for P3 + 7 d). The glutamate concentration was significantly decreased at P3 + 4 d and P3 + 7 d in a dose-dependent manner in the α-asaronol-treated rats compared with the NS-treated rats; however, there was no obvious change in glutamate concentration at P3 + 12 h (Table 1). Meanwhile, HI-induced activation of the ionotropic glutamate receptor AMPA receptor (AMPAR) was confirmed by a p-GluR2 (Tyr876) increase, and α-asaronol treatment decreased the level of p-GluR2 at P3 + 12 h, P3 + 4 d, and P3 + 7 d (Figure 3).

**TABLE 1:** The glutamate concentration was determined by HPLC in the ipsilateral hemisphere at different time points after HI. Establishment of a standard curve of the glutamate standard. The linear range is from 1 to 200 μg/ml. Correlation coefficient: *r* = 0.9996. All data are expressed as the mean ± SEM. ***p < 0.001 vs. sham. ^*#*^ *p* < 0.05, ^*##*^ *p* < 0.01, ^*###*^ *p* < 0.001 vs. HI + NS. *N* = 3–5 for each group.

Group	Sham	HI + NS	HI + *α*-asaronol		
Data			25 mg/kg	50 mg/kg	100 mg/kg
P3 + 12 h	0.198 ± 0.019	0.341 ± 0.029^***^	0.296 ± 0.016	0.265 ± 0.020	0.259 ± 0.031
P3 + 4 d	0.176 ± 0.014	0.402 ± 0.076^***^	0.268 ± 0.012^#^	0.246 ± 0.017^##^	0.205 ± 0.015^###^
P3 + 7 d	0.174 ± 0.019	0.364 ± 0.007^***^	0.244 ± 0.016^#^	0.215 ± 0.013^##^	0.204 ± 0.004^##^

All data are expressed as the mean ± SEM. *N* = 3–5. ***p < 0.001 vs*.* sham. ^*#*^ *p <* 0.05, ^*##*^ *p <* 0.01, ^*###*^ *p <* 0.001 vs. HI + NS.

### α-Asaronol Increases the Expression of GLT-1 and PPARγ in Astrocytes of Preterm White Matter Injury Rats

**FIGURE 4:**
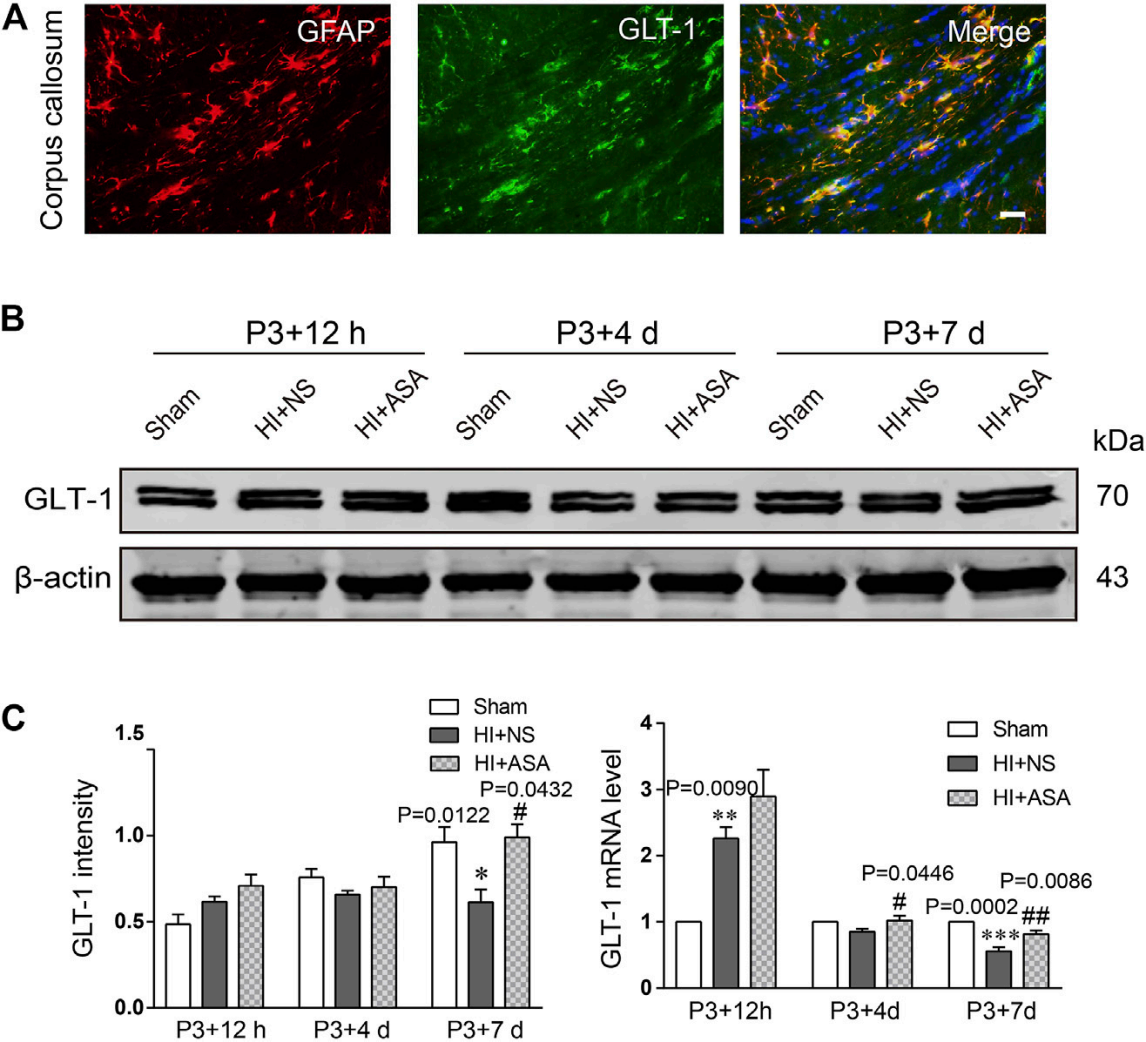
*α*-Asaronol increases the expression of GLT-1 in the PWMI rat model. **(A)** GLT-1 protein expression was detected by western blotting at P3 + 12 h, P3 + 4 d, and P3 + 7 d. **(B)** GLT-1 was quantified and normalized against β-actin. **(C)** The mRNA level of GLT-1 was quantified by qPCR. All data are represented as the mean ± SEM. *p < 0.05, **p < 0.01, ***p < 0.001 vs. sham group, ^#^ *p* < 0.05, ^##^ *p* < 0.01 vs. HI + NS group. *N* = 4 for each group.

Astrocytic GLT-1 is mainly responsible for the clearance of glutamate. We explored whether the decreased concentration of glutamate in PWMI rats treated with α-asaronol is mediated by GLT-1. The location of GLT-1 on astrocytes was confirmed by GLT-1/GFAP double labeling (Figure 4A). Data from the western blot and RT-qPCR revealed that GLT-1 levels were mildly increased at P3 + 12 h but significantly decreased at P3 + 7 d in the NS group compared with the sham group. α-Asaronol treatment obviously increased GLT-1 protein and mRNA levels at P3 + 7 d (Figures 4B,C).

**FIGURE 5:**
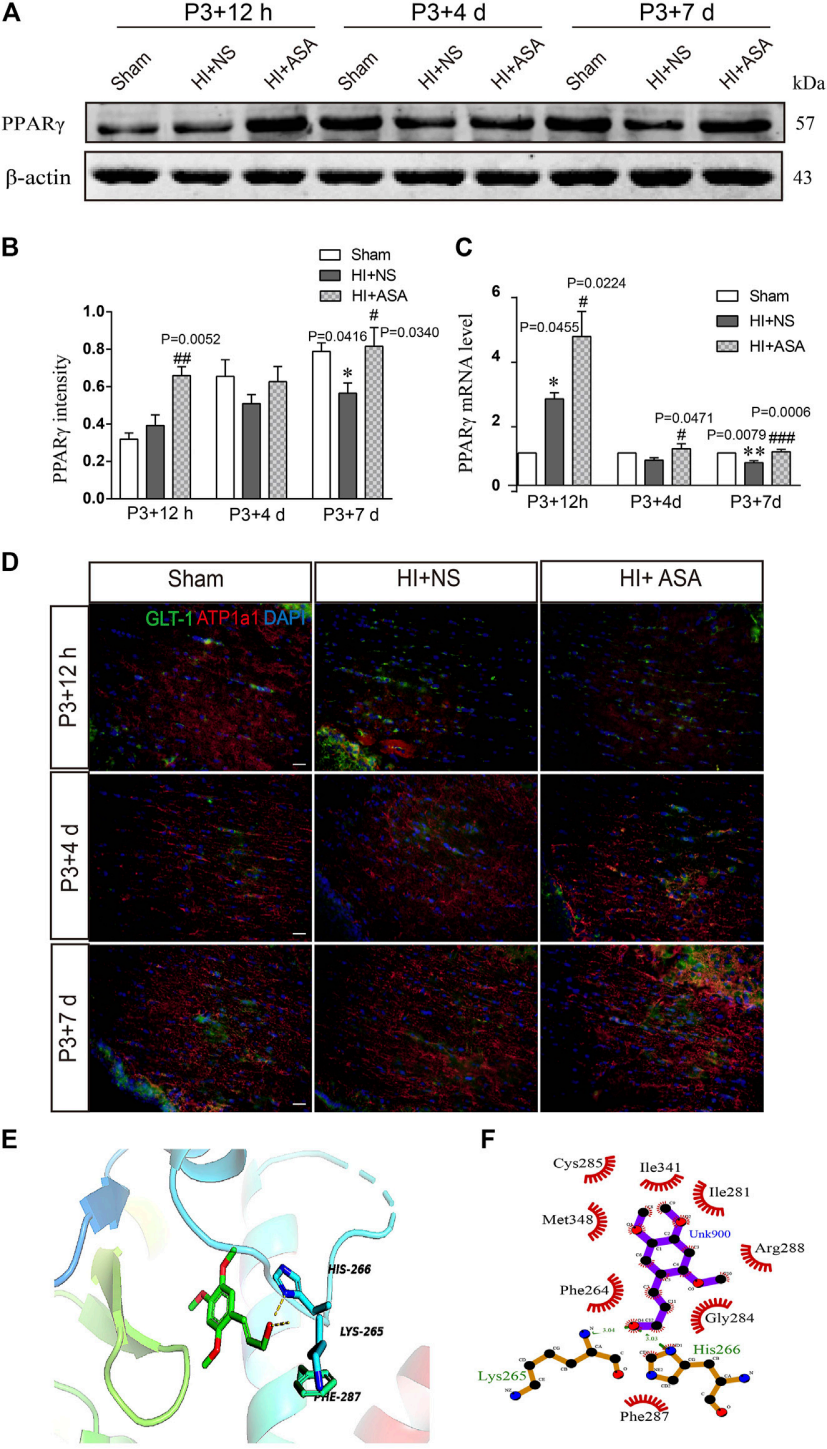
*α*-Asaronol increases the expression of PPARγ in PWMI models. **(A)** Expression of PPARγ was detected at P3 + 12 h, P3 + 4 d, and P3 + 7 d after HI. **(B)** PPARγ was quantified and normalized against *β*-actin. **(C)** The mRNA level of PPARγ was quantified by qPCR. **(D,E)** Representative immunofluorescence images for GLT-1 and ATP1A1 double staining in the corpus callosum. Scale bar, 20 µm. **(F,G)** Molecular docking simulation of α-asaronol and PPARγ. All data are represented as the mean ± SEM. *p < 0.05, **p < 0.01 vs. sham group, ^#^ *p* < 0.05, ^###^ *p* < 0.001 vs. HI + NS group. *N* = 4 for each group.

As one of the target genes of PPARγ, GLT-1 is regulated by PPARγ during the acquisition of brain ischemic tolerance (Romera et al., 2007) and is involved in neuroprotection by increasing glutamate uptake in ischemic preconditioning rats (Zhao et al., 2019). Our previous study also found that α-asaronol could promote the mRNA expression of PPARγ in zebrafish (Jin et al., 2020). Thus, we speculated that α-asaronol upregulates the expression of GLT-1, which may be dependent on PPARγ activation. As expected, we identified highly upregulated PPARγ protein and mRNA levels at P3 + 7 d in PWMI rats treated with α-asaronol (Figures 5A–C).

In addition, because the transport function of GLT-1 relies on transmembrane Na^+^/K^+^ gradients, we further examined the expression of ATP1α1, the alpha-1 subunit of a protein pump known as a Na^+^/K^+^ ATPase. At P3 + 12 h after HI, the fluorescence intensity of ATP1α1 was much weaker in the NS group than in the sham group. Although the α-asaronol treatment did not alter the expression of ATP1α1 at P3 + 12 h, ATP1α1 expression gradually returned to basal levels in PWMI rats treated with α-asaronol at P3 + 4 d and P3 + 7 d (Figure 5D).

A molecular docking simulation was performed to investigate the potential binding of α-asaronol and PPARγ. The Surflex-Dock molecular docking program was used to evaluate the results through various scoring functions, and the total score was calculated by valuing the polarity, hydrophobicity, enthalpy, and salvation. The higher the score, the more stable the docking complex. In the docking with PPARγ, the total score of α-asaronol was 5.523. As shown in Figure 5E, the α-asaronol molecule entered the binding pocket of PPARγ. α-Asaronol combined with the PPARγ residues LYS295, PHE264, MET348, CYS285, ILE341, ILE281, ARG288, GLY284, and HIS266 *via* van der Waals interactions. Furthermore, the hydroxyl group at the C12 position interacts with LYS265 and HIS266 on PPARγ to form hydrogen bonds. The methoxyl groups at the C1, C2, and C4 sites interact with PPARγ to form hydrophobic interactions and play a role in localization. The ethyl side chain at the C5 position extends into the hydrophobic cavity composed of PHE264 and GLY284 (Figure 5F). These results suggested that α-asaronol may activate PPARγ through physical interaction.

### α-Asaronol Increases the Expression of Glutamate Transporter 1 in Astrocytes Under Oxygen and Glucose Deprivation Conditions

**FIGURE 6:**
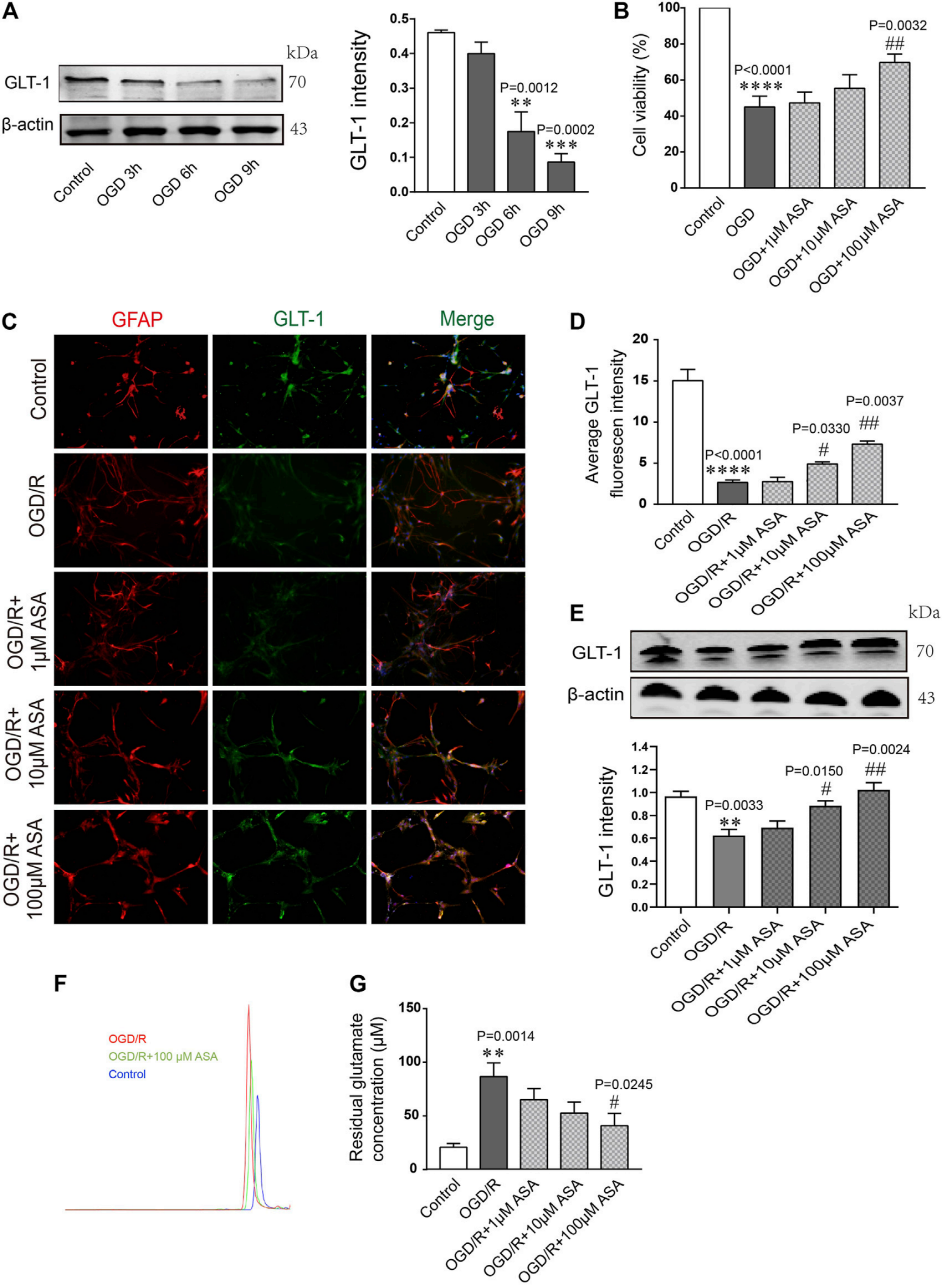
*α*-Asaronol increases the expression of astrocytic GLT-1 under OGD/R conditions. **(A)** Gradient OGD/R conditions of astrocytes. **(B)** Cell viability of astrocytes. **(C)** Representative immunofluorescence images for GFAP and GLT-1 staining in the corpus callosum for all groups. Scale bar, 20 µm. **(D)** Statistical analysis of GLT-1 mean fluorescence intensity. **(E)** The expression of GLT-1 was detected and quantified. **(F,G)** Chromatographs of glutamate and quantitative analysis of glutamate concentration in the medium for all groups were performed. All data are represented as the mean ± SEM. **p < 0.01, ****p < 0.0001 vs. the control group, ^#^ *p* < 0.05, ^##^ *p* < 0.01 vs. the OGD/R group. *N* = 4 for each group.

Astrocytes were subjected to OGD for 3, 6, or 9 h and then reoxygenated for 12 h. Quantification of the western blot data showed that the level of GLT-1 was obviously reduced at OGD 6 h compared with the control and OGD 3 h; thus, OGD 6 h was chosen for the following research experiments (Figure 6A). Astrocytes were pretreated with different doses of α-asaronol and then subjected to OGD for 6 h and reoxygenation for 12 h. CCK-8 data showed that 100 µM α-asaronol improved astrocyte viability (Figure 6B). In addition, 100 µM α-asaronol also significantly enhanced the average fluorescence intensity of GLT-1 (Figures 6C,D) and the protein level (Figure 6E). More interestingly, the glutamate concentration in the medium was lower in the 100 µM α-asaronol group than in the OGD group, as determined by HPLC (Figures 6F,G).

### α-Asaronol Upregulated Glutamate Transporter 1 Expression in Astrocytes by Activating PPARγ

**FIGURE 7:**
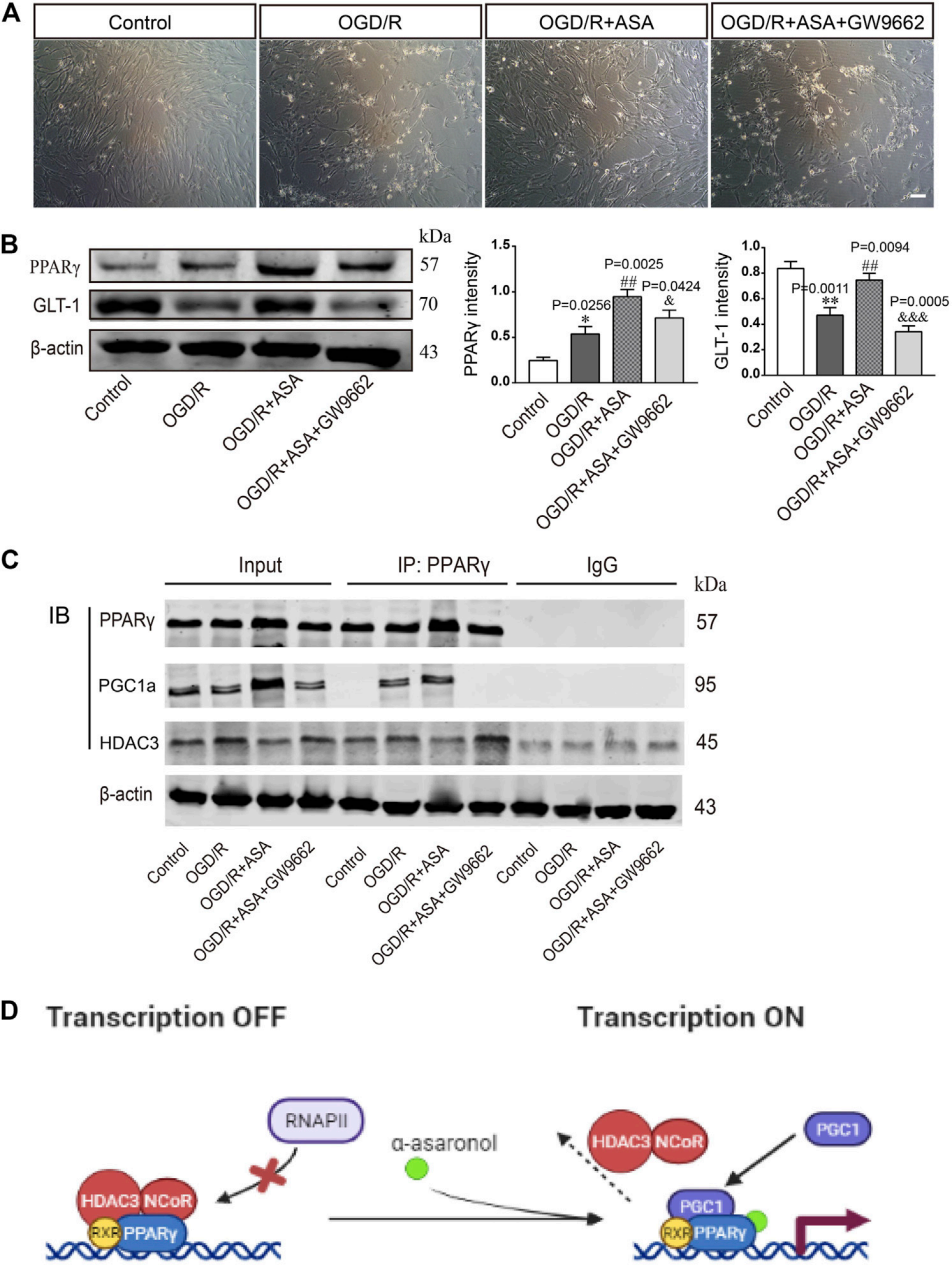
*α*-Asaronol upregulated GLT-1 expression in astrocytes by activating PPARγ. **(A)** The morphology of astrocytes under the bright field. Scale bar, 50 µm. **(B)** The expression of PPARγ and GLT-1 was detected by western blotting. PPARγ and GLT-1 were quantified and normalized against *β*-actin. **(C)** Co-IP was used to examine the interaction of PPARγ and PGC1a or PPARγ and HDAC3. **(D)** Graphical image representing the ligand-independent repression and α-asaronol-dependent transactivation of PPARγ. All data are represented as the mean ± SEM. *p < 0.05, **p < 0.01 vs. control group, ^##^ *p* < 0.01 *vs.* OGD/R group, ^&^ *p* < 0.05, ^&&&^ *p* < 0.001 vs. OGD/R + ASA group. *N* = 4 for each group.

The PPARγ selective antagonist GW9662 was used to study whether α-asaronol upregulates astrocyte GLT-1 by activating PPARγ. First, we noticed that OGD/R led to some of the astrocytes detaching from the culture dish and clustering, and α-asaronol treatment alleviated the morphological changes of astrocytes. However, the effect of α-asaronol on astrocyte morphology was reversed by GW9662 treatment (Figure 7A). Second, GW9662 also inhibited the increase in GLT-1 induced by α-asaronol (Figure 7B). Finally, we identified that OGD induced the interaction of PGC-1a and the PPARγ heterodimer, while GW9662 weakened the interaction by decreasing the level of PGC-1a, suggesting that OGD and α-asaronol treatment activated PPARγ signaling (Figures 7C,D).

### Conditioned Medium From α-Asaronol-Treated Astrocytes Promotes Oligodendrocyte Precursor Cell Differentiation Without Influencing Apoptosis

**FIGURE 8:**
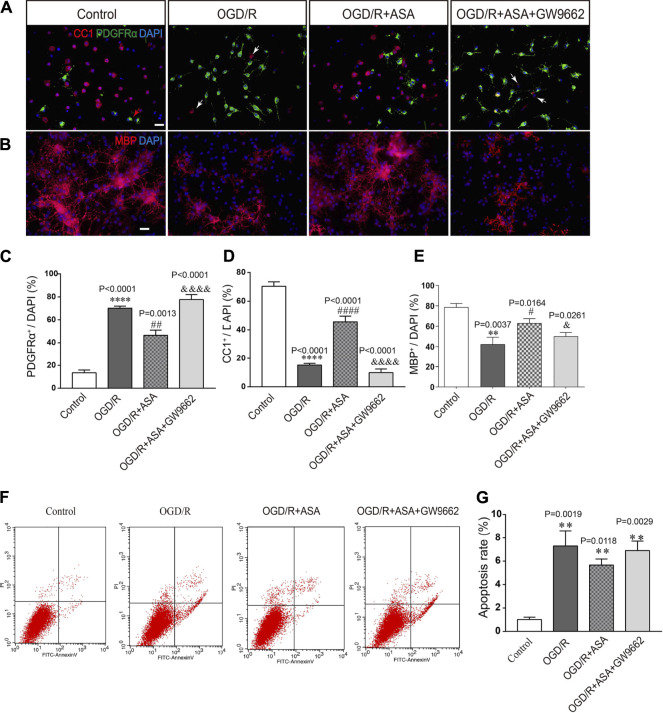
Conditioned medium from *α*-asaronol-treated astrocytes promotes OPC differentiation without influencing apoptosis. Representative immunofluorescence images for PDGFRα-labeled OPCs and CC1-labeled mature OLs **(A)** and MBP-labeled myelin sheath **(B)**. Scale bar, 20 µm. **(C–E)** Statistical analysis of the numbers of PDGFRα^+^ cells, CC1^+^ cells, and MBP^+^ cells. **(E,F)** Representative flow cytometry images of OPCs and quantitative analysis of the percentage of apoptotic cells for all groups. Scale bar, 20 µm. All data are represented as the mean ± SEM. **p < 0.01, ****p < 0.0001 vs. the control group. ^#^ *p* < 0.05, ^##^ *p* < 0.01, ^####^ *p* < 0.0001 vs. the OGD/R group. ^&^ *p* < 0.05, ^&&&&^ *p* < 0.001 vs. the OGD/R + ASA group. *N* = 4 per group.

The conditioned medium from astrocytes was collected and used to treat OPCs. Compared with the control group, the percentage of PDGFR*α* ^+^ cells was increased, while the percentages of CC1^+^ and MBP^+^ cells were significantly decreased after treatment with conditioned medium from OGD/R astrocytes. In contrast, the conditioned medium from OGD/R and *α*-asaronol cotreated astrocytes reduced the percentage of PDGFR*α* ^+^ cells and increased the percentage of CC1^+^ and MBP^+^ cells. However, the conditioned medium from GW9662-treated astrocytes reversed the effects of *α*-asaronol on OPC differentiation (Figures 8A–D). The apoptosis rate of OPCs treated with OGD/R-conditioned medium was increased compared with that of the control group, but there were no obvious differences in the apoptosis rate of OPCs among the OGD/R, OGD/R + NS, and OGD/R+*α*-asaronol groups by flow cytometry (Figures 8E,F).

## Discussion

In the CNS, over-activating glutamate receptors damage OPCs, and the subsequent differentiation disorder has always attracted attention from researchers (Back et al., 2007). In the present study, our data showed that the concentration of glutamate in the brains of PWMI rats was significantly higher than that in the brains of sham rats. Treatment with α-asaronol significantly decreased the concentration of glutamate, increased GLT-1 levels, promoted OPC differentiation, and alleviated dysmyelination. The neuroprotective mechanism of α-asaronol may be related to the increase in glutamate uptake by activating PPARγ-GLT-1 signaling in astrocytes.

Dysmyelination or demyelination is mainly due to failure of OPC differentiation rather than OPC depletion (Billiards et al., 2008; Buser et al., 2012). Studies have reported that the total pool of OPCs increases severalfold within 24 h after HI and continues to increase for a few days. Although numerous OPCs are regenerated in lesions, they fail to differentiate into myelinating oligodendrocytes following established procedures (Segovia et al., 2008; Wright et al., 2010). Consistent with previous studies, we also found that PDGFR*α*-labeled OPCs sharply increased in the acute phase of HI in neonatal rats; however, dysmyelination of the corpus callosum was still obvious at P3 + 4 d and P3 + 7 d. Interestingly, α-asaronol treatment promoted the differentiation of OPCs into myelinating oligodendrocytes and myelination. Studies have indicated that glutamate excitotoxicity is responsible for neuronal death in acute neurological disorders. When neurons from the rat cortex were exposed to different concentrations of glutamate (10–2,000 µM), glutamate induced a concentration-dependent increase in neuronal death. However, astrocytes exhibited a protective function in neuronal damage induced by glutamate (Zhang et al., 2019). Meanwhile, a high concentration of glutamate or activation of the ionotropic glutamate receptor AMPAR also causes oligodendrocyte precursor excitotoxicity (Deng et al., 2006), inhibiting the proliferation and differentiation of OPCs (Fannon et al., 2015). Reducing extracellular glutamate levels obviously improved HI-induced PWMI in neonatal rats (Yeh et al., 2017). Thus, we speculated that the effect of α-asaronol on myelination may be related to the clearance of glutamate.

The pharmacokinetics of *a*-asaronol and its main metabolite in rats were estimated by HPLC in our previous study (Sun et al., 2019). Tissue distribution evaluation showed that α-asaronol is distributed rapidly and widely in various tissues in the order of brain > heart > kidney > spleen > liver > lung and is eliminated quickly. The maximal concentration of α-asaronol in the brain is approximately 1.603 ± 0.221 μg/g at 5 min, and the half-life of α-asaronol is approximately 6 h. Unfortunately, in this study, we did not examine the half-life of α-asaronol in the HI-related part of the brain, which should be taken into consideration and clarified in future studies.

Glutamate transporters play a crucial role in the efficient clearance of glutamate (Magi et al., 2019). EAAT2 (rodent ortholog GLT-1) is the predominant glutamate transporter in the forebrain and carries out more than 95% of glutamate clearance (Zhou et al., 2014). It has been reported that GLT-1 and ATP-α interact within the processes of astrocytes (Rose et al., 2009; Roberts et al., 2014), and the maintenance of low extracellular glutamate levels depends on ATP-α activation and Na^+^ homeostasis (Pellerin and Magistretti, 1997). The maternal separation resulted in early disrupted neuron-glia integrity and axon-myelin entities in the rat brain, as evidenced by inhibited myelination, lowered expression of GLT-1 and ATP-α, and increased glutamate (Zeng et al., 2020). In this study, a drastic increase in glutamate concentration was accompanied by a high level of GLT-1 within 12 h after HI; however, the level of ATP1α1 in the model rats was lower than that in the sham rats, suggesting that the glutamate transport function of GLT-1 was poor at the acute phase of HI. At P3 + 4 d and P3 + 7 d, the glutamate concentration was still high, but the expression of GLT-1 was decreased in PWMI rats, while α-asaronol enhanced the levels of GLT-1 and ATP1α1 and decreased the glutamate concentration, suggesting that α-asaronol promoted glutamate uptake by upregulating GLT-1. *In vitro*, our study showed that GLT-1 protein expression was downregulated gradually after OGD/R in astrocytes, and 100 µM α-asaronol upregulated GLT-1 expression and enhanced glutamate uptake, indicating that the glutamate-scavenging capacity was increased in astrocytes because α-asaronol upregulated the functional expression of astrocytic GLT-1.

As a transcription factor of the nuclear hormone receptor superfamily, PPARγ plays a neuroprotective role in various CNS diseases (Schintu et al., 2009; Zhao et al., 2009; San et al., 2015). It has been reported that the PPARγ agonist rosiglitazone decreases infarct volume and increases GLT-1 expression after cerebral ischemia (Romera et al., 2007). Importantly, our previous research found that α-asaronol attenuated pentylenetetrazol-induced seizures in zebrafish and activated PPARγ (Jin et al., 2020). In the present study, both GLT-1 and PPARγ expression were upregulated in PWMI rats with α-asaronol treatment. To explore the mechanism by which α-asaronol regulates GLT-1 expression, we performed Surflex-Dock molecular docking analyses. The higher total score of α-asaronol docking with PPARγ indicated that α-asaronol and PPARγ could possibly form a stable docking complex.

PPARγ activation is a ligand-dependent process. In the absence of ligands, the PPARγ heterodimer is associated with corepressors, such as nuclear receptor corepressors and HDACs. This complex binds to the peroxisome proliferator response element in the promoter region of target genes and retains the genes in a suppressed state. Following ligand binding, the heterodimer dissociates from corepressors and recruits coactivators such as PGC1a, thereby upregulating target gene transcription (Cai et al., 2018; Bernardo et al., 2021). Here, our results showed that OGD and α-asaronol treatment led to PGC1a being recruited to the PPARγ heterodimer, while HDAC3 dissociated from the heterodimer, suggesting that OGD and α-asaronol treatment activated PPARγ signaling.

In our previous study, we examined the effects of different concentrations of glutamate receptor subunit 3 peptide B antibodies (GluR3B Abs), which can themselves activate GluR3-containing glutamate/AMPAR, on OPC viability. The results showed that a high concentration of GluR3B Ab induced OPC excitotoxicity by causing mitochondrial dysfunction (Liu et al., 2017). In this study, the conditioned medium from α-asaronol-treated astrocytes promoted OPC differentiation, and PPARγ antagonist GW9662 treatment not only reversed the effect of α-asaronol on OPC differentiation but also decreased the expression of GLT-1 and increased the concentration of glutamate, suggesting that PPARγ-GLT-1 mediated the effect of α-asaronol on OPC differentiation and myelination.

## Conclusion

Taken together, this study proved that α-asaronol can relieve the disorder of OPC differentiation and dysmyelination induced by glutamate excitotoxicity in a PWMI rat model by regulating PPARγ-GLT-1 signaling. Unfortunately, whether α-asaronol binds to PPARγ directly or indirectly was not investigated in this study. In the future, yeast two-hybrid systems, cellular thermal shift assays, or surface plasmon resonance techniques should be used to study α-asaronol and PPARγ interactions.

## Data Availability

The original contributions presented in the study are included in the article/Supplementary Material, further inquiries can be directed to the corresponding authors.
